# Efficacy of neuroendoscopic and small-bone-window craniotomy microsurgery for hypertensive cerebral hemorrhage: a meta-analysis of Chinese RCT studies

**DOI:** 10.3389/fneur.2024.1434928

**Published:** 2024-08-30

**Authors:** Tianpeng Zhi, Hang Wang, Xiangyang Wei, Zhengjun Wei, Hong-tao Sun

**Affiliations:** ^1^Tianjin Key Laboratory of Neurotrauma Repair, Characteristic Medical Center of People’s Armed Police Forces, Tianjin, China; ^2^Emergency surgery, Tianjin First Central Hospital, Tianjin, China

**Keywords:** supratentorial hypertensive intracerebral hemorrhage, neuroendoscopic surgery, small-bone-window craniotomy, efficacy, meta-analysis

## Abstract

**Objective:**

To compare the clinical efficacy of neuroendoscopy and small-bone-window craniotomy microsurgery in the treatment of supratentorial hypertensive intracerebral hemorrhage.

**Methods:**

A search was conducted for Chinese randomized controlled trials on neuroendoscopy and small-bone-window craniotomy microsurgery treatment of hypertensive intracerebral hemorrhage published before February 1, 2024, in PubMed, Embase, Web of Science, Cochrane Library, China National Knowledge Infrastructure, Wanfang Data, and China Science and Technology Journal Database. Meta-analysis was performed using Review Manager 5.4 software.

**Results:**

We included 9 randomized controlled trials, with 391 cases in the neuroendoscopy group and 403 cases in the craniotomy group. The meta-analysis results showed that compared to the small-bone-window craniotomy group, the neuroendoscopy group had a higher rate of hematoma clearance (95% CI [6.65, 18.52], *p* < 0.00001), less intraoperative bleeding (95% CI [−294.83, −284.75], *p* < 0.00001), shorter operation time (95% CI [−138.65, −63.04], *p* < 0.00001), fewer days in the ICU (95% CI [−8.56, −4.04], *p* < 0.00001), lower rate of postoperative complications (95% CI [0.15, 0.50], *p* < 0.0001), lower NIHSS score at 3 months postoperatively (95% CI [−6.82, −5.36], *p* < 0.00001), and higher ADL score (95% CI [16.5, 20.07], *p* < 0.00001). All comparison results were statistically significant.

**Conclusion:**

Compared with small-bone-window craniotomy microsurgery, neuroendoscopic surgery for episodic hypertensive cerebral hemorrhage resulted in a higher rate of hematoma clearance, less intraoperative bleeding, shorter operative time, fewer days in the ICU, a lower rate of postoperative complications and a lower 3-month postoperative NIHSS score, and a higher ADL score.

## 1 Introduction

Hypertensive Intracerebral Hemorrhage (HICH) is one of the serious complications of hypertension and a common neurosurgical disorder characterized by high incidence rates (1, 2), high mortality, and high disability rates (3). Hypertensive intracerebral hemorrhage is a destructive hemorrhagic condition caused by the rupture of small intracranial vessels due to high blood pressure, leading to bleeding into the brain parenchyma. It most commonly occurs as supratentorial hemorrhage, accounting for 80% of all hypertensive intracerebral hemorrhage cases, severely impacting daily life and human health (4, 5). Currently, the key to treating patients with supratentorial hypertensive intracerebral hemorrhage lies in the early removal of the hematoma, alleviation of mass effect, reduction of intracranial pressure, and early restoration of function in compressed neurons to improve prognosis (6, 7). Surgical treatments for hypertensive intracerebral hemorrhage include decompressive craniectomy with hematoma evacuation, minicraniotomy microsurgery, directed drainage, minimally invasive neuroendoscopic surgery, and stereotactic hematoma aspiration (8).

Neuroendoscopy and small-bone-window craniotomy microsurgery, as emerging treatment modalities, have gradually attracted attention. Neuroendoscopic surgery, using microscopic techniques, accesses the intracranial hemorrhage area to remove the hematoma, alleviating intracranial pressure and improving cerebral circulation, thereby relieving the symptoms and complications of ICH. Small-bone-window craniotomy surgery, on the other hand, creates a cranial window to extract the hematoma while also reducing intracranial pressure and minimizing secondary brain injury. Although neuroendoscopy and Small-bone-window craniotomy surgery have a theoretical basis and clinical prospects, their efficacy has not yet been fully confirmed. Some single-center clinical studies indicate that both surgical techniques have a positive impact on the treatment of ICH, improving patients’ neurological functions and quality of life. However, due to limitations such as small sample sizes and less rigorous study designs, these results lack generalizability and reliability. Therefore, this article utilizes evidence-based medicine methods to perform a meta-analysis on the efficacy and safety of minimally invasive neuroendoscopic surgery and Small-bone-window craniotomy surgery in treating supratentorial hypertensive cerebral hemorrhage, aiming to provide references for the selection of surgical methods in clinical practice.

## 2 Materials and methods

### 2.1 Inclusion and exclusion criteria

#### 2.1.1 Inclusion criteria

(1) The studies included are randomized controlled trials; (2) The included patients meet the diagnostic criteria for hypertensive cerebral hemorrhage consistent with the “Chinese Multidisciplinary Diagnosis and Treatment Guidelines for Hypertensive Cerebral Hemorrhage” (9), “Guidelines for the Treatment of Cerebral Hemorrhage” (10), or the 2022 update of the American Heart Association/American Stroke Association Guidelines for the Management of Spontaneous Intracerebral Hemorrhage (11), and with clinical diagnosis indicating bleeding in the cerebral lobe and basal ganglia region.

#### 2.1.2 Exclusion criteria

(1) Non-randomized controlled studies; (2) Patients with hemorrhage caused by reasons other than hypertension; (3) Studies without available data; (4) Studies with unclear inclusion or exclusion criteria for patients; (5) Conferences, guidelines, animal studies, etc.; (6) Duplicate publications by the same researcher or institution.

### 2.2 Surgical strategy

#### 2.2.1 Neuroendoscopy group

Patients are placed under general anesthesia in a supine position with the head elevated and feet lowered on the operating table. Using neuronavigation, the location and volume of the hematoma are identified, and the puncture site, angle, and depth are determined. A linear incision approximately 4 cm in length is made along the midline parallel to the incision point, centering on the puncture site. The scalp is cut and retracted, a bone window about 2 cm in diameter is created, and the dura mater is cut in a cross-shaped manner. Cortical vessels are avoided, and local cortical coagulation is performed using bipolar electrocoagulation.

A disposable minimally invasive brain surgery cannula is inserted according to the preoperative design for puncture site, angle, and depth. Once the puncture reaches the hematoma cavity, the inner core is removed, and the hematoma is observed and completely cleared using a neuroendoscope. Hemostasis is achieved by covering the site with absorbable gelatin sponge. Routine drainage is performed using a drainage tube, and the surgical incision is closed layer by layer.

#### 2.2.2 Small-bone-window craniotomy group

Under general anesthesia, the patient’s scalp is incised to locate the thickest part of the hematoma nearest to the skin. A curved or straight incision, 9–15 cm in length, is made sequentially through the skin and the galea aponeurotica. The pericranium is incised and stripped along the inner edge of the incision. The skull is perforated with an electric drill, and then the cranial bone is milled open with a milling cutter, creating a bone flap measuring 3 cm × 4 cm to 5 cm × 6 cm. The dura mater is incised. Under a microscope, the cortex is incised approximately 1 cm to create an opening to the hematoma, exposing the surgical field adequately. The hematoma is evacuated and electrocauterization is performed for hemostasis. After confirming no bleeding points upon hematoma clearance, the dura mater is sutured, and a silicone drainage tube is left outside the dura mater. The bone flap is replaced, and the surgical incision is closed layer by layer, followed by disinfection of the incision site.

### 2.3 Outcome indicators

The primary outcome measure is the hematoma clearance rate (the percentage difference between preoperative hematoma volume and postoperative residual hematoma volume relative to preoperative hematoma volume); secondary outcomes include surgery duration, intraoperative blood loss, ICU stay duration, National Institute of Health Stroke Scale (NIHSS) score, complications such as pulmonary and intracranial infections and Activities of Daily Living score (ADL) (12). The ADL score ranges from 0 to 100 points. Scores are categorized as follows: 0–20 points indicate complete dependence in daily living; 21–40 points indicate requiring extensive assistance in daily living; 41–60 points indicate needing some assistance in daily living; 61–80 points indicate being mostly independent in daily living; and 81–100 points indicate complete independence in daily living.

### 2.4 Search strategy

Searches were conducted independently by two researchers in databases including PubMed, Embase, Web of Science, Cochrane Library, China National Knowledge Infrastructure, Wanfang Data, and China Science and Technology Journal Database, with the search extending to February 1 2024. Search terms included “Cerebral hemorrhage,” “hypertensive cerebral hemorrhage,” “supratentorial hypertensive cerebral hemorrhage,” “basal ganglia,” “neuroendoscopy,” “small bone window,” “surgery.”

### 2.5 Data extraction

Potential studies were collected from literature databases and imported into EndNote to remove duplicates. Two researchers then independently excluded ineligible studies by reviewing titles and abstracts based on the inclusion and exclusion criteria. Full texts of the remaining studies were read to determine the inclusion of the studies. Discrepancies were resolved through consultation with a third researcher. Key data, including characteristics of the included studies (first author, year of publication, sample size), patient demographics (age, gender), intervention measures, and outcome indicators (primary and secondary outcomes), were independently extracted by two researchers and input into Excel. Information on risk of bias assessment (randomization, allocation, measurement, and follow-up bias) was also extracted.

### 2.6 Risk of bias assessment

The risk of bias was assessed using the Cochrane “Risk of Bias” tool (13), which is used to evaluate several important biases in clinical trials, such as selection bias, implementation bias, measurement bias, follow-up bias, and reporting bias. The risk of bias was judged independently by two researchers as low, high, or unclear. Discrepancies were resolved through consultation or by consulting with a third researcher.

### 2.7 Statistical analysis

The effects of continuous variables and dichotomous variables were estimated using odds ratios (ORs) with 95% confidence intervals (CIs). Meta-analysis was conducted using Review Manager 5.4 software, employing a fixed-effect model (I2 < 50%) or a random-effects model (I2 > 50%) as appropriate. A *p*-value < 0.05 was considered statistically significant.

## 3 Results

### 3.1 Literature search

The initial search yielded 285 studies. After removing 98 duplicates, 76 studies unrelated to the research objective, and 17 items such as journal catalogs and conference reports, titles and abstracts were reviewed to exclude 31 non-randomized controlled studies and 14 studies with interventions that did not meet the criteria. This left 48 studies for preliminary screening. After full-text review, 40 studies that did not meet the inclusion criteria were excluded, resulting in 9 studies being included in the statistical analysis (14–21).

### 3.2 Characteristics of included studies

**Figure 1:**
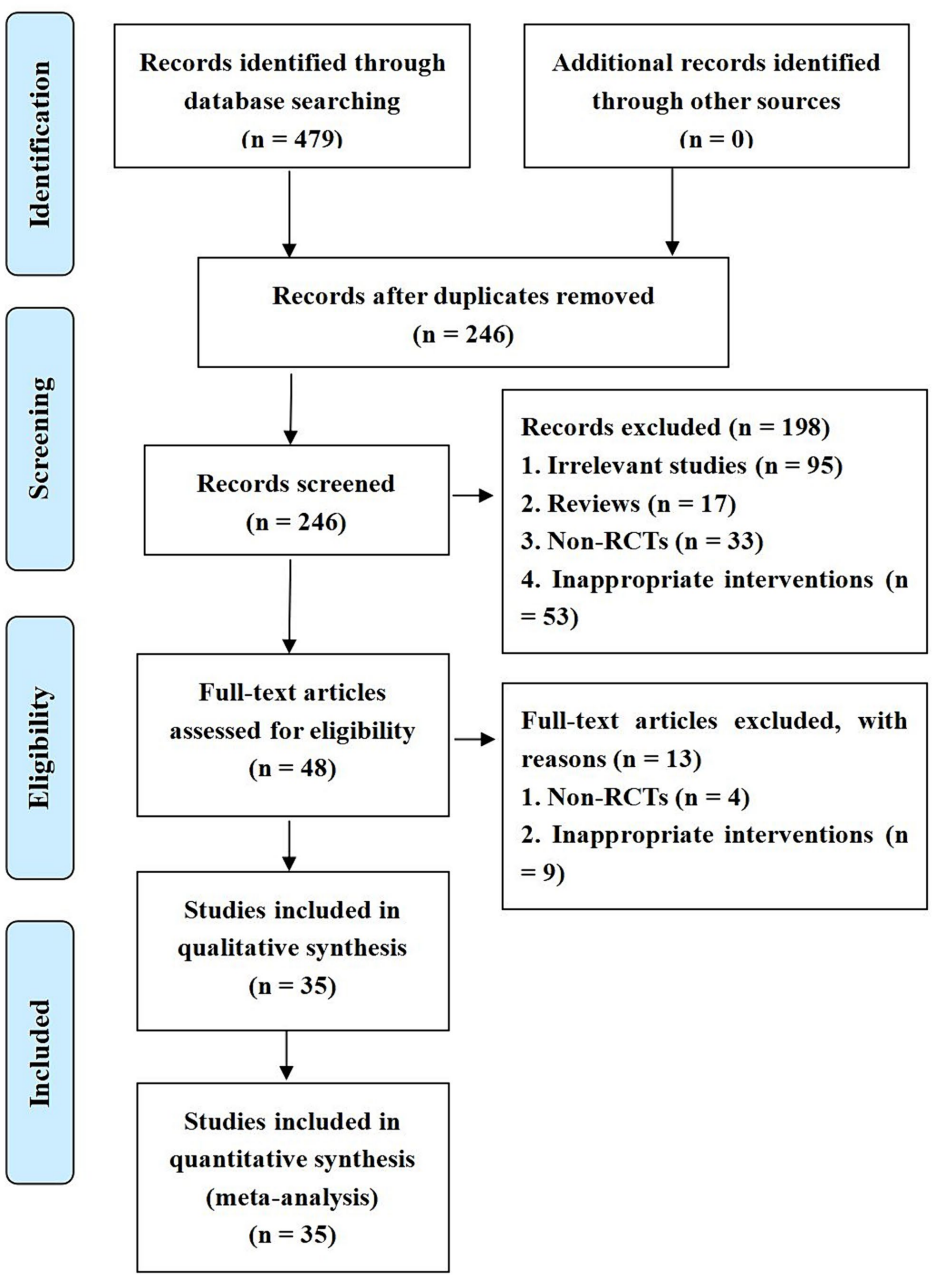
A flowchart for research literature inclusion.

The inclusion of the study involved 794 patients, 391 in the neuroendoscopy group and 403 in the small bone window group, and the inclusion process is shown in Figure 1. The sample sizes for both the experimental and control groups ranged from 29 to 50 patients. Relevant data were extracted independently by two professionals in the field, and the characteristics of the included studies are detailed in Table 1.

**Table 1:** Baseline data of included studies.

Author	Hematoma location	Number of patients (E/C)	Male (E/C)	Female (E/C)	Age (E/C) (岁)	Hematoma volume mL (E/C)	Experimental group	Control group	Outcome measures
Gu et al. (13)	Basal Ganglia	45/45	25/24	20/21	55.90 ± 4.79/55.90 ± 4.79	53.19 ± 10.15/52.57 ± 10.23	Neuroendoscopy	Small bone window	①②③④
Shi (14)	Lobe of the brain	50/50	28/29	22/21	50.1 ± 1.5/49.8 ± 2.8	–	Neuroendoscopy	Small bone window	①②③④⑥
Mei and Xu (15)	Lobe of the brain	40/40	23/24	17/16	60.0 ± 2.6/59.3 ± 2.4	–	Neuroendoscopy	Small bone window	①②③④⑥
Shi (16)	Lobe of the brain	45/45	23/21	22/24	55.31 ± 3.84/55.28 ± 3.25	30–50	Neuroendoscopy	Small bone window	①②③④
Zhang and Yang (17)	Basal Ganglia	48/48	26/25	22/23	57.19 ± 11.69/57.52 ± 10.73	52.10 ± 3.74/52.14 ± 3.72	Neuroendoscopy	Small bone window	①②③⑤
Shen (18)	Lobe of the brain	42/42	26/24	16/18	55.28 ± 2.09/55.30 ± 2.04	51.76 ± 13.71/51.76 ± 13.72	Neuroendoscopy	Small bone window	①②③④⑤⑥
Zhou and Zhang (19)	Basal Ganglia	34/34	19/20	15/14	50.61 ± 11.45/49.89 ± 10.27	53.19 ± 10.15/52.57 ± 10.23	Neuroendoscopy	Small bone window	①⑤⑥
Liu (20)	Lobe of the brain	29/29	15/16	14/13	57.79 ± 6.37/57.62 ± 6.12	44.91 ± 5.38/44.84 ± 5.27	Neuroendoscopy	Small bone window	①②③④⑥
Lv et al. (22)	Basal Ganglia	58/70	37/48	21/22	56.74 ± 13.69/54.76 ± 12.62	–	Neuroendoscopy	Small bone window	①②③④⑥

E, Experimental group; C, Control group; ①: Hematoma clearance rate; ②: Intraoperative blood loss; ③: Surgery duration; ④: ICU stay duration; ⑤: NIHSS score; ⑥: Complications.

### 3.3 Risk of bias assessment

The results of the risk of bias assessment are shown in Figure 1. Eight studies were assessed as having a low risk of bias in the generation of random sequences because they reported specific methods for generating random sequences. All included studies were classified as having a low level of follow-up bias due to complete outcome data. Due to a lack of sufficient information, the risks of bias for allocation concealment, implementation, and measurement in all included studies were rated as unclear.

### 3.4 Comparison of hematoma clearance rates between neuroendoscopic surgery and small-bone-window craniotomy surgery

**Figure 2:**
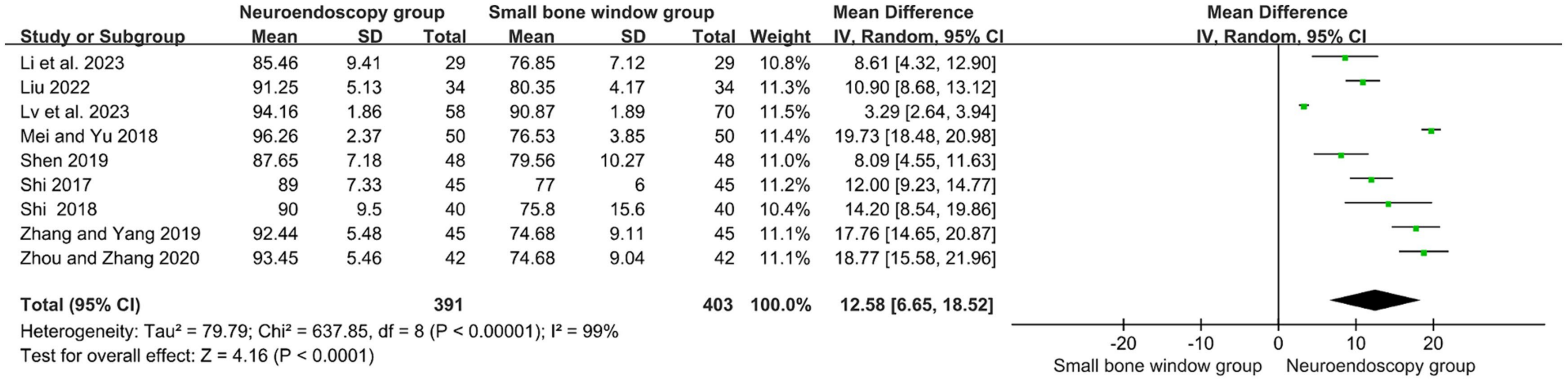
Forest plot and meta-analysis of hematoma clearance rates of neuroendoscopic surgery and minicraniotomy surgery.

A total of nine randomized controlled studies assessed hematoma clearance rates in neuroendoscopic and small-bone window craniotomy microsurgery. High heterogeneity (I2 = 99%), and therefore Meta-analysis using a random-effects model, as shown in Figure 2, MD = 12.58, 95% CI [10.24, 17.48], *p* < 0.00001, indicated that hematoma clearance was higher in neuroendoscopic than in small-bone-window craniotomy microsurgery.

### 3.5 Comparison of intraoperative blood loss between neuroendoscopic surgery and small-bone-window craniotomy surgery

**Figure 3:**
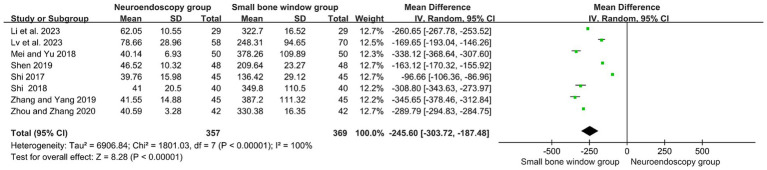
Forest plot and meta-analysis of the rate of intraoperative blood loss in neuroendoscopic surgery and minicraniotomy surgery.

Eight randomized controlled studies assessed intraoperative bleeding (mL) in neuroendoscopic and small-bone-window craniotomy microsurgery. As shown in Figure 3, with high heterogeneity (I2 = 100%), Meta-analysis was performed using a random-effects model. The results showed MD = −245.60, 95% CI [−303.72, −167.48], *p* < 0.00001, indicating that intraoperative hemorrhage was significantly less in neuroendoscopic surgery than in small-bone-window craniotomy microsurgery.

### 3.6 Comparison of surgery duration between neuroendoscopic surgery and small-bone-window craniotomy surgery

**Figure 4:**
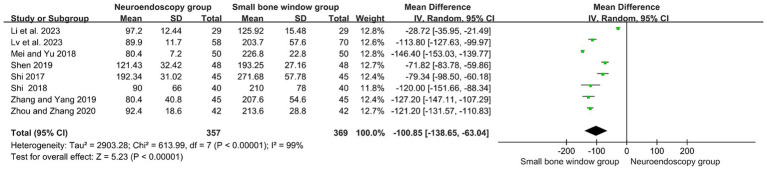
Forest plot and meta-analysis of surgery duration for neuroendoscopic surgery and minicraniotomy surgery.

A total of eight randomized controlled studies assessed the operative time (min) for neuroendoscopic and small-bone-window craniotomy microsurgery. High heterogeneity (I2 = 99%), and therefore Meta-analysis using a random-effects model, as shown in Figure 4, MD = −100.85, 95% CI [−138.65, −63.04], *p* < 0.00001, suggests that the operative time used for neuroendoscopic surgery was significantly lower than that used for small-bone-window craniotomy microsurgery.

### 3.7 Comparison of ICU stay duration after neuroendoscopic surgery and small-bone-window craniotomy surgery

**Figure 5:**
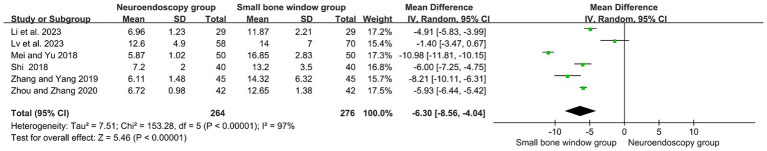
Forest plot and meta-analysis of ICU stay duration after neuroendoscopic surgery and minicraniotomy surgery.

Six randomized controlled studies assessed the duration (in days) of ICU admission after neuroendoscopic and small-bone-window craniotomy microsurgery. As shown in Figure 5, high heterogeneity (I2 = 97%), and therefore Meta-analysis using a random-effects model with MD = −6.03, 95% CI [−8.56, −4.04], *p* < 0.00001, indicated that the number of days of ICU stay after neuroendoscopic surgery was significantly reduced compared with that of small-bone-window craniotomy microsurgery.

### 3.8 Comparison of NIHSS scores after neuroendoscopic surgery and small-bone-window craniotomy surgery

**Figure 6:**
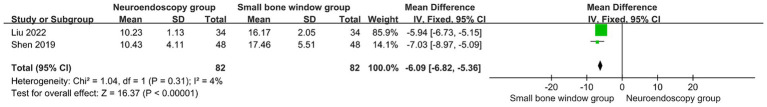
Forest plot and meta-analysis of NIHSS scores after neuroendoscopic surgery and minicraniotomy surgery.

Two randomized controlled trials evaluated NIHSS at 3 months post neuroendoscopy versus Small-bone-window craniotomy surgery. The results showed I2 = 4%, thus a fixed-effects model was used for meta-analysis, MD = −6.09, 95% CI [−6.82, −5.36], *p* < 0.00001 (Figure 6). Compared with the Small-bone-window craniotomy surgery group, the neuroendoscopy group had lower NIHSS scores, indicating a significant statistical difference between the two groups. This suggests that neuroendoscopic surgery causes less neurological damage than Small-bone-window craniotomy surgery.

### 3.9 Comparison of complications after neuroendoscopic surgery and small-bone-window craniotomy surgery

**Figure 7:**
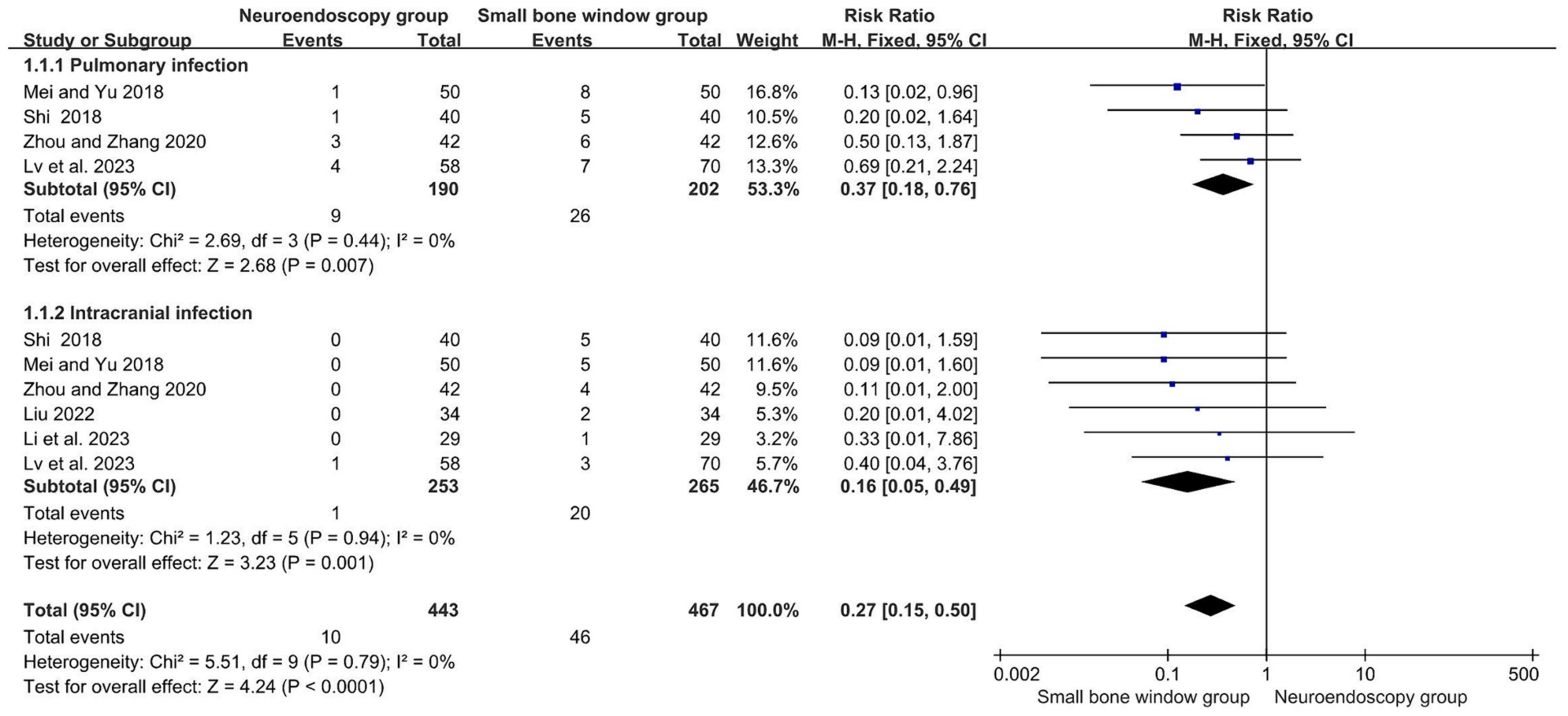
Forest plot and meta-analysis of complications after neuroendoscopic surgery and minicraniotomy surgery.

A total of six randomized controlled studies evaluated complications after neuroendoscopic and small-bone-window craniotomy microsurgery, with subgroup analyses based on different complications (pulmonary and intracranial infections). The results showed no heterogeneity between studies (I2 = 0%), so a fixed-effects model was used for Meta-analysis. As shown in Figure 7, MD = 0.27, 95% CI [0.15, 0.50], *p* < 0.0001, indicating that the complication rate after neuroendoscopic surgery was significantly lower than that of open cranial microsurgery with a small bone window.

### 3.10 Comparison of ADL scores after neuroendoscopic surgery and small-bone window craniotomy microsurgery

**Figure 8:**
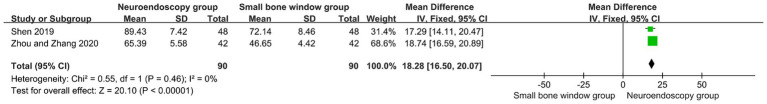
Forest plot and meta-analysis of comparison of ADL scores after neuroendoscopic surgery and minicraniotomy surgery.

Two randomized controlled studies assessed ADL scores 3 months after neuroendoscopic and small-bone window craniotomy microsurgery. The results showed I2 = 0%, so Meta-analysis using a fixed-effects model, 95% CI [−6.82, −5.36], *p* < 0.00001 (Figure 8), higher ADL scores in the neuroendoscopic group compared with the small-bone window craniotomy group, with a statistically significant difference between the two groups of patients. It indicated that patients with neuroendoscopic surgery recovered better than small bone window craniotomy microsurgery.

## 4 Discussion

Hypertensive cerebral hemorrhage accounts for 10–30% of all strokes, and most survivors have severe neurological deficits (23–25). Hypertensive cerebral hemorrhage can be divided into supratentorial and infratentorial types, with the former being more common. For supratentorial hypertensive cerebral hemorrhage, rapid removal of the hematoma can improve the cerebral hemorrhage condition, prevent ongoing compression of brain neural tissue, and alleviate brain injury (26, 27). Minicraniotomy microsurgery is relatively simple to perform, allows clear observation of the bleeding point in the brain, and enables quick control of the hematoma and bleeding. However, this approach causes significant brain trauma and often leads to complications such as intracranial infections and cerebral edema after surgery, posing considerable risks (28, 29).

In recent years, technological advancements have led to the emergence of new surgical methods in clinical practice for the treatment of hypertensive intracerebral hemorrhage, such as neuroendoscopic minimally invasive surgery and stereotactic localization-directed hematoma evacuation. Neuroendoscopic minimally invasive surgery is an accurate, simple to perform, effective, non-craniotomy, and blood transfusion-free surgical treatment method. It combines neuro-navigation, intraoperative ultrasound technology, and micro-neurosurgical techniques. With a small trauma and high hematoma clearance rate, this surgical system’s positioning principle is based on a digital three-dimensional coordinate system, where the location of any point in space can be determined by a three-dimensional directional coordinate system. By combining the vertical principle of the sagittal, horizontal, and coronal planes, a single focus can be achieved, and the patient’s head can be placed in a three-dimensional directional coordinate system, combined with the stereoscopic parameters provided by CT scans to accurately locate the lesion tissue. Neuroendoscopic minimally invasive surgery allows for panoramic exposure of the surgical field, avoiding residual hematoma, and enabling doctors to identify active bleeding points in high-definition three-dimensional images, thereby improving hemostatic efficiency, reducing intraoperative bleeding, and lowering the risk of re-bleeding. Currently, minimally invasive neuroendoscopic surgery mainly employs rigid endoscopes for operation. There are reports of using flexible endoscopes in the treatment of chronic subdural hematoma (30), but their application in intracerebral hematoma evacuation is limited. Rigid endoscopes can safely be inserted into the hematoma cavity to maximize hematoma clearance, reduce intraoperative bleeding, protect neural pathways, and aid in postoperative neurological function recovery. However, treatment guidelines have not yet recommended neuroendoscopy for treating supratentorial hypertensive cerebral hemorrhage, possibly due to the small number of clinical cases and a lack of large-scale prospective studies (31, 32). Therefore, it is still necessary to choose the appropriate surgical method based on the patient’s condition to enhance treatment effectiveness and promote early recovery.

Hematoma clearance rate is a key indicator for assessing the effectiveness of surgical treatment for hypertensive cerebral hemorrhage. Subgroup analysis of the MISTIE III (33) trial demonstrated that a reduction in clot size to 15 mL or less was associated with better mRS scores in patients at 365 days. Effective hematoma clearance can reduce compression on surrounding brain tissue, lower the risk of secondary brain injury, and thus improve clinical outcomes for patients. Additionally, rapid and thorough removal of the hematoma helps to reduce the development of cerebral edema and alleviate increased intracranial pressure, ultimately aiding in the restoration of neurological function. Intraoperative blood loss is an important indicator for evaluating surgical risk and patient recovery. Reducing blood loss during surgery can significantly decrease the risk of postoperative complications, especially reducing the likelihood of vascular accidents and infections. Furthermore, reducing bleeding also helps shorten recovery time, speeding up the patient’s return to normal life. Postoperative complications are one of the crucial metrics for assessing the safety and effectiveness of surgery. Fewer complications not only improve the patient’s quality of life but also reduce long-term medical costs. The reduction in complications is often closely associated with the choice of surgical method, the precision of the surgical operation, and the optimization of intraoperative management.

The results of this meta-analysis indicate that compared to minicraniotomy microsurgery, neuroendoscopic surgery for supratentorial hypertensive cerebral hemorrhage shows higher hematoma clearance rates, less intraoperative blood loss, shorter surgery times, fewer ICU days, a lower incidence of postoperative complications, and lower NIHSS scores at 3 months post-operation. However, the included studies were rated as unclear for risks of allocation concealment, implementation bias, and measurement bias; and the studies originated from various hospitals across the country, influenced by regional differences in medical standards and the surgeons’ expertise, leading to considerable heterogeneity in the results.

## 5 Conclusion

Compared with small-bone-window craniotomy microsurgery, neuroendoscopic surgery for episodic hypertensive cerebral hemorrhage resulted in a higher hematoma clearance rate, less intraoperative bleeding, shorter operative time, fewer days in the ICU, a lower rate of postoperative complications and a lower 3-month postoperative NIHSS score, and a higher ADL score. These findings provide a valuable reference for clinical practice and may drive more clinical applications to adopt neuroendoscopic techniques.

## Data Availability

The original contributions presented in the study are included in the article/supplementary material, further inquiries can be directed to the corresponding author.
